# Hepatitis C virus promotes hepatocellular carcinogenesis by targeting TIPE2, a new regulator of DNA damage response

**DOI:** 10.1007/s13277-016-5409-z

**Published:** 2016-09-30

**Authors:** Yaohui Wang, Yinan Jiang, Jinxue Zhou, Wuhui Song, Jing Li, Mingli Wang, Jiuge Chen, Rui Xu, Jingjing Zhang, Fanni Ma, Youhai H. Chen, Yuanfang Ma

**Affiliations:** 1 Henan Key Laboratory of Engineering Antibody Medicine, Medical College of Henan University, Kaifeng, Henan 475004 China; 2 College of Basic Medical Sciences, Zhengzhou University, Zhengzhou, Henan 450001 China; 3 Zhengzhou University Affiliated Tumor Hospital, Zhengzhou, Henan 450001 China; 4 Key Laboratory of Medical Molecular Virology, School of Basic Medical Sciences, Shanghai Medical College, Fudan University, Shanghai, China; 5 Department of Pathology and Laboratory Medicine, University of Pennsylvania Perelman School of Medicine, Philadelphia, PA 19104 USA

**Keywords:** Hepatitis C virus, Hepatocellular carcinoma, NS5A, TIPE2, DNA damage response

## Abstract

Infection of hepatitis C virus (HCV) is associated with primary hepatocellular carcinoma (HCC). However, its underlying molecular mechanisms remain enigmatic. Tumor necrosis factor-α-induced protein 8-like 2 (TIPE2), a new negative regulator of immunity, plays significant roles in modulating inflammation and tumorigenesis. We hypothesized that TIPE2 might be involved in the development of HCV-induced HCC. To test this hypothesis, the expression of TIPE2 was determined by Western blot in the tumor and pericarcinomatous tissues collected from ten HCV-positive HCC patients; the interaction between TIPE2 and HCV-encoded non-structural proteins was analyzed by immunoprecipitation and immunofluorescence assays, and tumorigenesis and its mechanisms were studied in cell models and nude mice. Our results demonstrated that the expression of TIPE2 was significantly reduced in HCC tissues compared to that in the paracarcinoma tissues. HCV-encoded non-structural protein NS5A could specifically interact with TIPE2 and induce its degradation. Downregulation of TIPE2 by shRNA in cell lines increased genomic DNA damage and promoted cell colony formation in vitro and tumorigenesis in nude mice. In contrast, overexpression of TIPE2 had an opposite effect. Downregulation of TIPE2 by NS5A is associated with genomic DNA instability and HCV-induced HCC development. Thus, TIPE2 may be a new therapeutic target for the treatment of HCV-associated HCC.

## Introduction

Currently, there are more than 150 million hepatitis C virus (HCV)-infected individuals in the world [1]. Chronic HCV infection is highly associated with hepatocellular carcinoma (HCC). HCC is the most common histological subtype of primary liver carcinoma and accounts for 70–85 % of total liver cancers [1, 2], and almost half of the patients are from China [3].

HCV, a sense-strand RNA virus, replicates exclusively in the cytoplasm and is unable to integrate into the host genome; the underlying mechanisms of HCV-induced hepatocarcinogenesis remain elusive. Among the ten HCV encoded structural (core, E1, E2) and non-structural (P7, NS2, NS3, NS4A, NS4B, NS5A, NS5B) proteins [4], core, NS3, NS4B, and NS5A have been shown to directly activate oncogenic molecular pathways and promote tumor formation in vivo [5–8]. The strategies employed by HCV and encoded proteins to induce tumor formation include chronic inflammation, reactive oxidative stress (ROS), steatosis, fibrosis, and so on. DNA damage/repair is associated with almost all of the above pathogenic patterns. In fact, core, NS3/4A, NS5A, and NS5B have been reported to enhance DNA damage or suppress damage repair [9–13]. Consistently, in HCC patients, accumulation of DNA damage has been detected in the peripheral blood lymphocytes [14] and abundant H2AX^+^ T lymphocytes were found in the liver [15].

Tumor necrosis factor-α-induced protein-8 like-2 (TIPE2 or TNFAIP8L2) is a newly identified protein essential for the maintenance of immune homeostasis [16]. The crystal structure of TIPE2 revealed a large hydrophobic central cavity as the binding sites for cofactors [17]. Apart from maintenance of immune homeostasis, TIPE2 inhibits Ras activity via binding RalGDS and thereafter suppresses Ras-induced tumorigenesis in mice [18]. Downregulation of TIPE2 is associated with poor prognosis of non-small cell lung cancer, and it can also inhibit HCC cell metastasis [19, 20]. A recent study showed that expression of TIPE2 was reduced in peripheral blood mononuclear cells and tumor tissues from HBV-infected patients compared to healthy individuals [21]. Interestingly, TIPE2 was also shown to negatively regulate oxidative burst, indicating a possible involvement of DNA damage in the TIPE2-mediated tumorigenesis [22]. However, whether TIPE2 and TIPE2-mediated DNA damage are involved in HCV-related HCC is still unknown.

In the present study, we investigated the association between TIPE2 and HCV-related HCC at clinical specimen, cell culture, and animal model aspects. The results showed that expression of TIPE2 is significantly reduced in tumor tissues compared to that in the paracarcinoma tissues from HCV-positive HCC patients. HCV/NS5A interacts with TIPE2 and promotes its degradation. Ectopic expression of TIPE2 can reduce DNA damages, while silencing TIPE2 with small hairpin (shRNA) can enhance it. Upregulation of TIPE2 can inhibit the HCC’s tumor characteristics. These results suggest that TIPE2 is a negative regulator of HCV-associated HCC.

## Materials and methods

### Clinical specimens

This study was approved by the Ethics Committees of the Medical College of Henan University, and written informed consent was obtained from all participants. Tumor and pericarcinomatous liver tissues were collected from ten HCV-positive HCC patients at the Affiliated Tumor Hospital of Zhengzhou University. The tissues were frozen at −80 °C, and protein was extracted by radioimmunoprecipitation (RIPA) tissue lysis buffer after grinding in liquid nitrogen. Patients’ clinical characteristics are presented in Table 1.

**Table 1:** Patients’ clinical characteristics in this study

	Patients
Number	10
Mean age (years)	65.6
Gender(male/female)	6**/**4
HCV (+)	10**/**10
HBV (+)	1**/**10
Mean AFP (ng/ml)	725.13

*AFP* Alpha-fetoprotein, *HBV* hepatitis B virus

### Animals and cells

The animal experiments performed in this study were prior approval by the Animal Experimentation Committee of Henan University. Four-week-old male BALB/cA-nu mice were purchased from Beijing HFK Bioscience. All mice received standard diet and water and were treated in accordance with the National Guide for the Care and Use of Laboratory Animals. The cell lines HEK293T, Changliver, and Huh7 cells were maintained in DMEM medium supplemented with 10 % fetal bovine serum, 2 mM l-glutamine, and penicillin (100 units/ml)-streptomycin (100 μg/ml).

### Antibodies and reagents

Mouse anti-Chk1, rabbit anti-Chk2, anti-H_2_AX, anti-phospho-Chk1, anti-phospho-Chk2, and anti γ-H_2_AX antibodies were purchased from Cell Signaling Technology (Danvers, USA). Mouse monoclonal anti-β-actin and anti-FLAG were purchased from Sigma (St. Louis, USA), and rabbit anti-TIPE2 was purchased from Boster (Wuhan, China). Puromycin, crystal violet, MTT, DMSO, and bovine serum albumin (BSA) were purchased from Sigma, and DMEM medium and fetal bovine serums were purchased from Gibco (Gran Island, USA). Lentiviral vector pLKO.1, psPAX2, and pMD2.G were obtained from Addgene.

#### Transfection and establishment of stable cell lines

For transient transfection, the cells were inoculated overnight and reached to 60–70 % confluence; a certain amount of plasmid DNA was transiently transfected into the cells with the X-tremeGENE HP DNA transfection reagent following the manufacturer’s protocol (Promega, Madison, USA). The cells were harvested 48 h post-transfection; they were subjected to the indicated experiments. To generate stable cell lines, the Changliver cells were transduced with lentiviral particles containing either TIPE2 shRNA or scramble shRNA. For ectopic expression of TIPE2, the Huh7 cells were infected with lentiviral particles expressing TIPE2 cDNA or empty vector. The cells were selected in 2 μg/ml puromycin for 2 weeks and were pooled for future study.

### Cell proliferation and plate colony formation assay

For plate colony formation assay, cells were detached and seeded in six-well plates with 500 cells per well in culture medium. Two weeks later, visible clone cell clusters appeared. The cells were washed twice with PBS and fixed in 4 % paraformaldehyde for 15 min followed by staining with crystal violet for 20 min. The cells were then washed with PBS and air-dried. The colony is quantified and photographed under microscopy. The colony number divided by total cell numbers accounts for the colony formation rate. For cell viability, the cells were seeded in 96-well plates at a density of 5 × 10^3^ cells per well, and cell growth curve was measured by MTT assay.

### DNA damage model in vitro

To construct DNA damage cell model, the cells were seeded into 12-well plates; 24 h later, culture medium was changed with fresh medium containing hydroxyurea or DMSO at 3 mM and incubated for another 24 h. Cell lysates were then collected for immune blotting analysis.

### Immunoblotting analysis and co-immunoprecipitation-

Cells were lysed with RIPA buffer containing protease inhibitor cocktail, and protein concentration was determined using bicinchoninic acid reagent (CWBIO, Beijing, China). Equal amounts of protein were loaded in each well for electrophoresis followed by transferring onto nitrocellulose filter membrane. After blocking with 5 % fat-free milk or 5 % BSA, the membranes were incubated with primary antibodies overnight at 4 °C. After washing in Tris-buffered saline and Tween (TBST) buffer for three times, the membranes were incubated with HRP-conjugated secondary antibody for 1 h at room temperature. Then, the membranes were washed in TBST for three to four times, and then, they were developed in ECL buffer for 2 min. The signals were measured by exposing to the X-ray film. For co-immunoprecipitation, the cells were lysed in immunoprecipitation (IP) lysis buffer containing protease and phosphatase inhibitors. About 1 mg proteins were incubated with proper primary antibody at 4 °C overnight (control IgG is used as negative control). Fifty microliters of 50 % slurry protein A-agarose beads was added to the cell lysates for 2 h. The protein A-agarose beads were pelleted and washed in TBST buffer for five times. The proteins that are eluted from protein A-agarose beads were subjected to the immunoblotting. For glutathione S-transferase (GST) pull-down assay, the cells were lysed in the IP lysis buffer. The equal amounts of lysated proteins were incubated with GST alone or GST-TIPE2 proteins that were purified from bacteria at 4 °C overnight. Fifty microliters of glutathione Sepharose 4b beads was added to the protein lysates and incubated for 1 h at 4 °C. The beads were pelleted and washed for five times in cell lysis buffer. The eluted proteins were subjected to the immunoblotting.

### Immunohistochemistry and immunofluorescence assay

For histology, tumor tissues were fixed with 4 % paraformaldehyde at 4 °C overnight for paraffin embedding. After dewaxing, the slides were incubated in 3 % H_2_O_2_ for 20 min to block endogenous peroxidase. Antigen retrieval was done at 95 °C for 20 min with citric acid hydrochloric acid antigen retrieval buffer. Tissue sections were incubated with blocking buffer containing 5 % BSA for 30 min and then subsequently with primary and secondary antibodies. SABC (BOSTER, Wuhan, China) was utilized to amplify signal before hematoxylin and AEC or DAB (ZSGB-BIO, Beijing, China) staining.

Cells grown on glass coverslips were analyzed with immunofluorescence assay as previous [13]. Briefly, the cells were fixed with 4 % paraformaldehyde and permeabilized with 0.4 % Triton X-100. After blocking with 3 % BSA, the cells were incubated with primary antibodies and subsequently with fluorescein isothiocyanate (FITC)-labeled secondary antibodies. Coverslips were mounted with Prolong Gold Anti-fade Reagent (containing DAPI, Thermo, Hudson, USA). The signals were observed and photographed under the confocal microscopy (Nikon, ECLIPSE Ti).

### Tumor models

Four-week-old male BALB/cA-nu mice were injected subcutaneously in the right underarms with Huh7 stable cells and Changliver stable cells (5 × 10^6^ cells per 100 μl). After inoculation, tumor growth was monitored daily for 2–4 weeks with a vernier caliper until the tumor size reached approximately 100 mm^3^. Mice were sacrificed by spinal cord dislocation for tumor collection and subsequent detection at indicated time.

### TUNEL staining

For TUNEL staining, mouse sh-TIPE2 tumor tissue and sh-Ctrl/TIPE2 Huh7 cells were fixed with 4 % paraformaldehyde, permeabilized with 0.1 % Triton X-100, and stained with TUNEL labeling solution (Roche, Switzerland) according to the manufacturer’s instructions.

## Results

### Downregulation of TIPE2 in tumor tissues of HCV-positive HCC

**Fig. 1:**
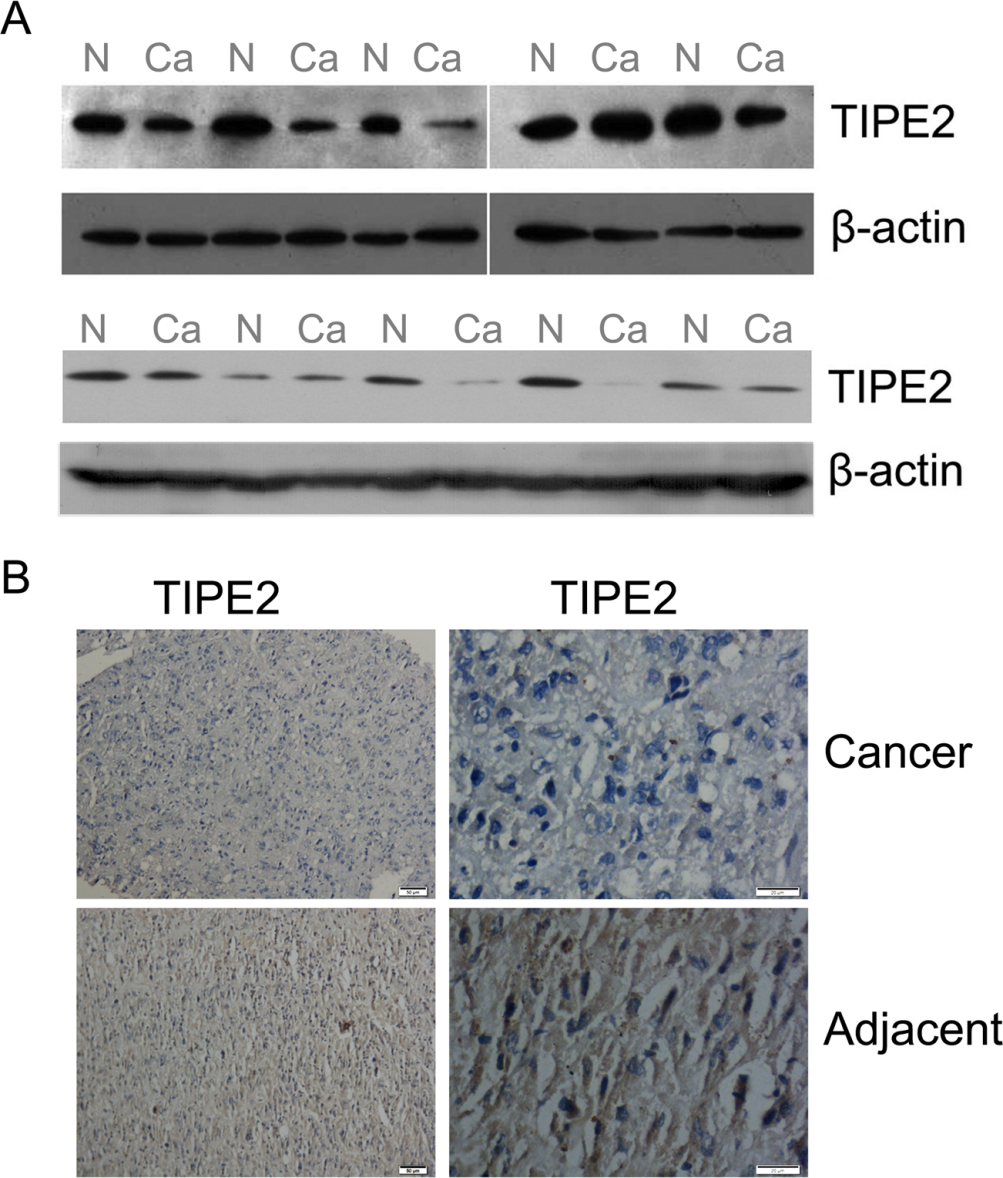
Downregulation of TIPE2 in HCV-positive HCC tumor tissues. Representative photographs of TIPE2 protein expression in HCV-positive HCC tissues (Ca) and adjacent liver tissues (N) examined by Western blot (**a**) and IHC (**b**) with anti-TIPE2 antibody. Positive cells were stained in *brown*. *Scale bars* = 200 μm

To study the roles of TIPE2 in HCV-associated HCC, the expressions of TIPE2 in the HCV-positive HCC tissues were measured by immunoblotting. Ten HCV-positive HCC and their paracarcinoma tissues were applied to immunoblotting. As indicated in Fig. 1, the expression of TIPE2 is significantly reduced in eight of ten HCV-positive HCC tissues compared to that in the tissues adjacent to the HCC tissues (Fig. 1a). To exclude the contamination of other tissues or immune cells, immunohistochemistry was performed to confirm the downregulation of TIPE2 in hepatocyte (Fig. 1b). Similarly, lower level of TIPE2 was observed in the hepatoma cell lines such as Huh7 and HepG2 cells compared to non-cancer Changliver cells (data not shown). These results suggested that TIPE2 is involved in HCV-associated HCC.

### HCV non-structural protein NS5A promotes TIPE2 degradation

**Fig. 2:**
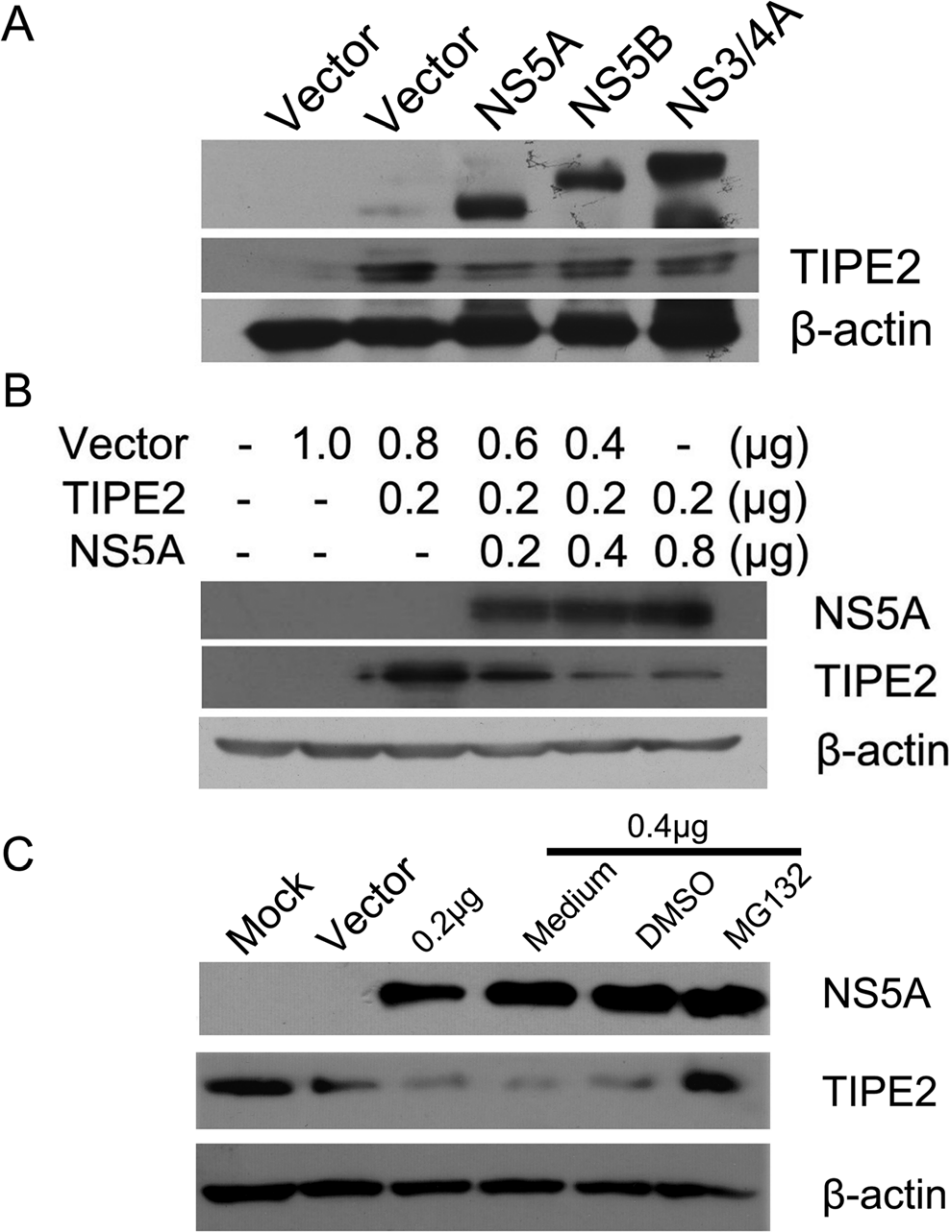
HCV-encoded NS5A leads to TIPE2 degradation. **a** HEK293T cells were incubated in 12-well plate; when the confluence is up to 70 %, cells were transiently co-transfected with TIPE2 (0.5 μg) and equal amount of HCV NS5A, NS5B, NS3/4A, or vector plasmids as indicated. Forty-eight hours post-transfection, expression of TIPE2 was determined by Western blot. **b** TIPE2 was co-transfected into 293T cells with increasing amounts of NS5A plasmids. Forty-eight hours later, cell lysates were subjected to SDS-PAGE and analyzed by Western blot. **c** TIPE2 was co-transfected into 293T cells with increasing amounts of NS5A plasmids. Cells were treated with DMSO or 1 mM MG132 6 h before harvesting. Forty-eight hours post-transfection, cell lysates were subjected to SDS-PAGE and analyzed by Western blot

HCV promotes HCC development via encoding a number of oncoproteins such as Non-structural proteins NS3/4A, NS5A, and NS5B. To determine whether downregulation of TIPE2 in HCV-positive HCC tissue (Fig. 1) is regulated by HCV non-structural proteins, TIPE2 was transiently co-expressed with NS3/4A, NS5A, or NS5B in 293T cells. The immunoblotting results indicated that NS5A could significantly reduce TIPE2 protein expression level (Fig. 2a) in a dose-dependent manner (Fig. 2b). Furthermore, the downregulation of TIPE2 could be reversed by proteasome inhibitor, MG132 (Fig. 2c), which suggested that the degradation of TIPE2 induced by NS5A is proteasome dependent.

### NS5A interacts with TIPE2

**Fig. 3:**
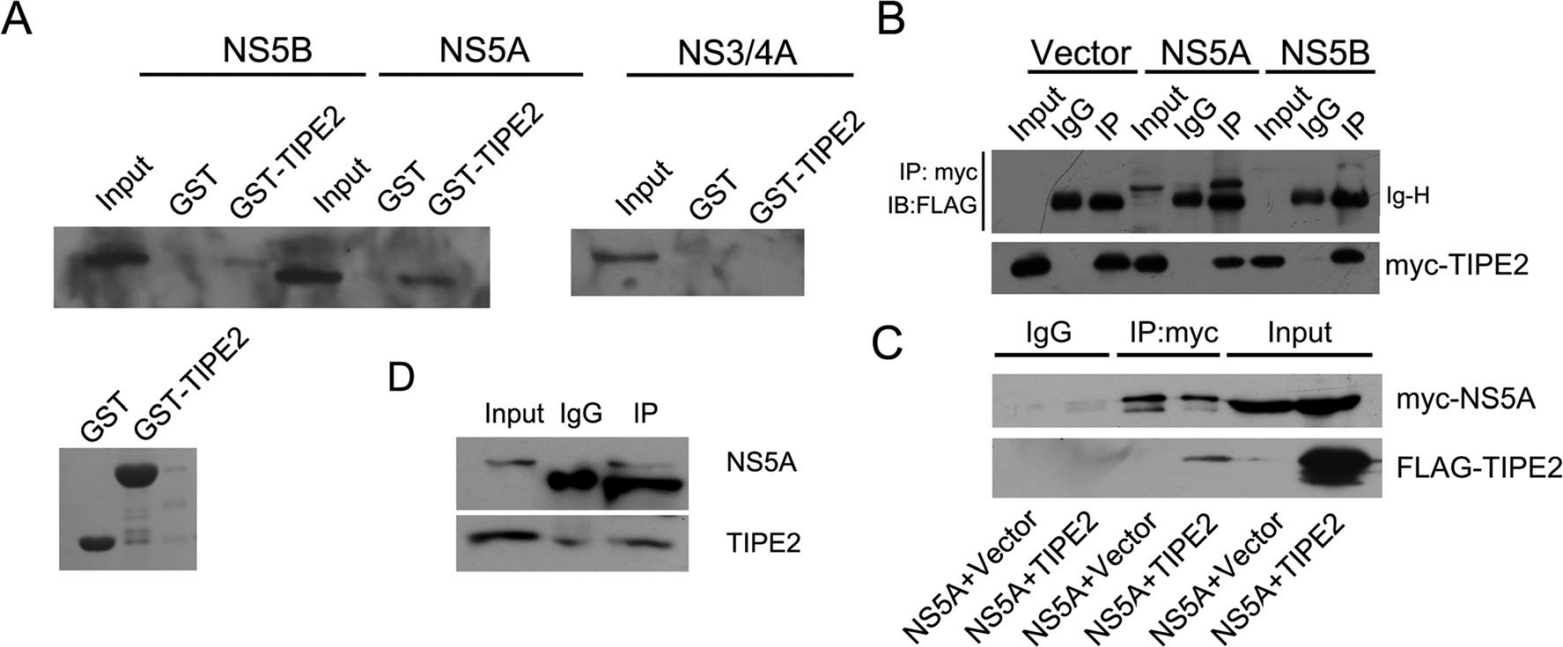
Interaction between TIPE2 and NS5A. **a** The association of TIPE2 with NS5A, NS5B, or NS3/4A was detected by GST pull-down assay, and Coomassie Brilliant Blue staining of TIPE2 expressed in prokaryotic expression cells (BL21) was shown. **b** Myc-TIPE2 was transiently co-transfected into 293T cells with FLAG-NS5A or FLAG-NS5B. Cell lysates were co-immunoprecipitated with anti-myc antibody or isotype IgG followed by Western blot. **c** 293T cells were transiently transfected with FLAG-TIPE2 and myc-NS5A. The cell lysates were co-immunoprecipitated with anti-FLAG antibody or isotype IgG followed by Western blot. **d** Huh7 TIPE2 stable cells were seeded into 10-cm dish and transfected with 6 μg myc-NS5A plasmids, and 48 h later, co-IP was performed as above

Since NS5A could reduce TIPE2 expression, we predict that there may be interactions between them. To test this hypothesis, we constructed GST and GST-TIPE2 expression plasmids and expressed them in prokaryotic BL21 cells. The GST pull-down assay (Fig. 3a) revealed that TIPE2 was strongly associated with NS5A but hardly with NS5B or NS3/4A (Fig. 3a).

To further confirm the interaction between TIPE2 and NS5A, co-IP of myc-TIPE2 and FLAG-NS5A with anti-FLAG antibody was performed. The result demonstrated a strong interaction between TIPE2 and NS5A (Fig. 3b). co-IP of FLAG-TIPE2 and myc-NS5A showed similar results (Fig. 3c). co-IP from Huh7 cells indicated that the interaction also exists in hepatoma cells (Fig. 3d).

**Fig. 4:**
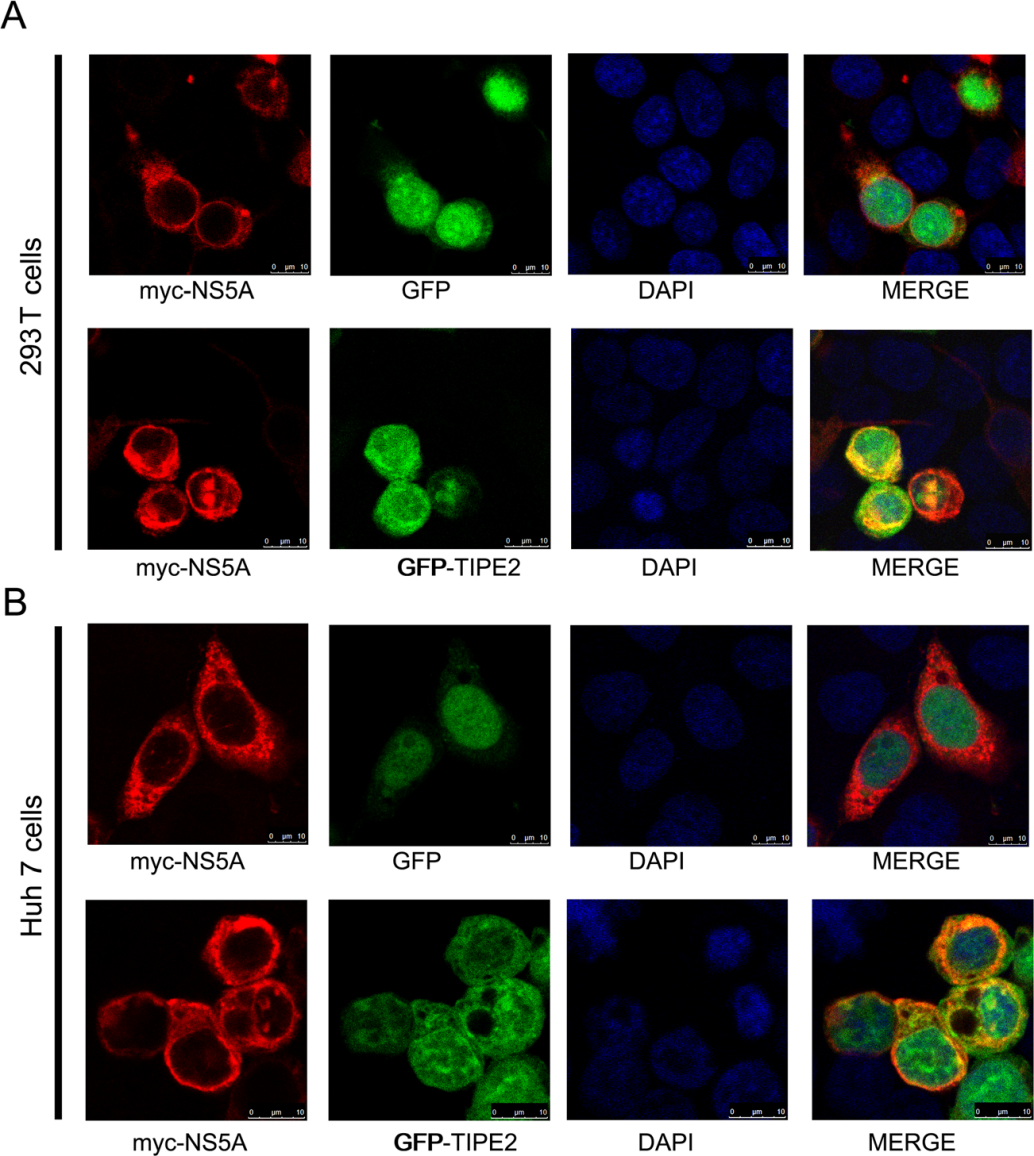
Co-localization of TIPE2 and NS5A in cytoplasm. Myc-NS5As were co-transfected into 293T cells (**a**) or Huh7 cells (**b**) with EGFP or EGFP-TIPE2 plasmids, respectively. After 48 h, the cells were fixed, stained, and analyzed with confocal microscopy

To visualize the co-localization of TIPE2 and NS5A in situ, we co-transfected GFP-TIPE2 and myc-NS5A plasmids into 293T cells. Confocal microscopy revealed an obvious co-localization of TIPE2 and NS5A in cytoplasm (Fig. 4a). The co-localization of TIPE2 and NS5A was also observed in Huh7 cells (Fig. 4b). Collectively, we concluded that NS5A binds TIPE2 and possibly leads to degradation of TIPE2.

### TIPE2 inhibits the DDR in vitro

**Fig. 5:**
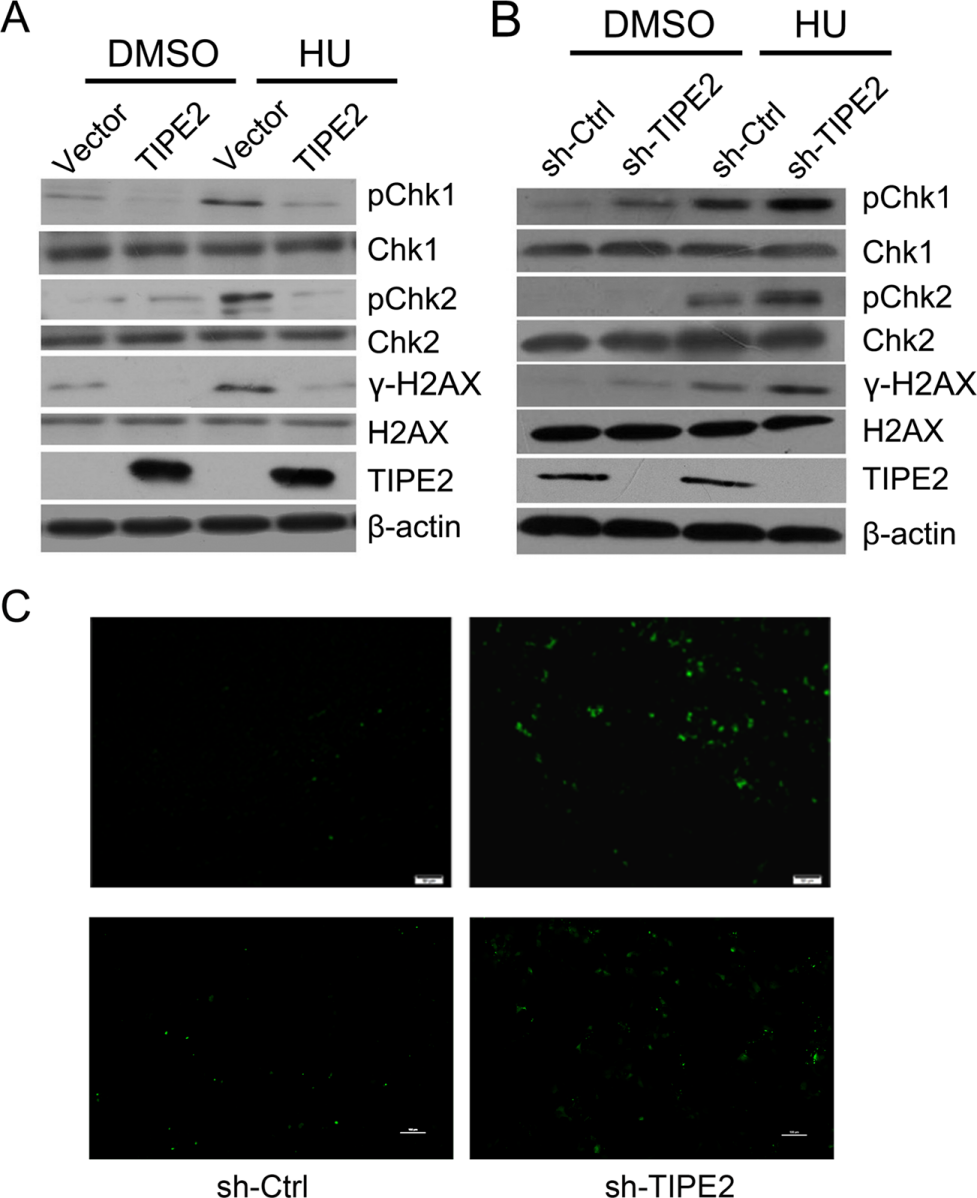
Downregulation of TIPE2 stimulates DNA damage response. **a** TIPE2-overexpressing stable cell line and control cell line were treated with hydroxyurea or DMSO as indicated. Cell lysates were collected and analyzed for phospho-H2AX, Chk1, and Chk2 levels with Western blot. **b** sh-TIPE2 and corresponding sh-Ctrl stable cell lines were exposed to hydroxyurea or DMSO, and then, the phospho-H2AX, Chk1, and Chk2 levels were determined by Western blot. **c** TUNEL staining was performed in mice sh-TIPE2 tumor tissue and Huh7 sh-TIPE2 stable cells. *Green*: TUNEL-positive cells

Cancer development is accompanied with continuous DNA damage. We therefore speculated that TIPE2 might be involved in DNA damage/repair. To determine whether TIPE2 is involved in tumor’s DNA damage/repair, we generated Huh7-TIPE2-overexpressing stable cell line and Changliver-TIPE2 knockdown stable cell line by using lentivirus infection. The cells were left without treatment or treated with hydroxyurea (HU) for inducing DNA damage. The results indicated that ectopic expression of TIPE2 could reduce the phosphorylation level of H2AX, Chk1, and Chk2 in Huh7-TIPE2 cells (Fig. 5a). In contrast, the phosphorylation level of H2AX, Chk1, and Chk2 was much elevated in Changliver-TIPE2 knockdown cells (Fig. 5b). Consistently, we also observed the increase of TUNEL-positive cells in mice sh-TIPE2 tumor tissue and Huh7 stable cells (Fig. 5c). These results indicate that TIPE2 directly induces DNA damage and diminishes HU-induced DNA damage response.

### TIPE2 suppressed hepatocellular tumorigenesis in vitro and in vivo

**Fig. 6:**
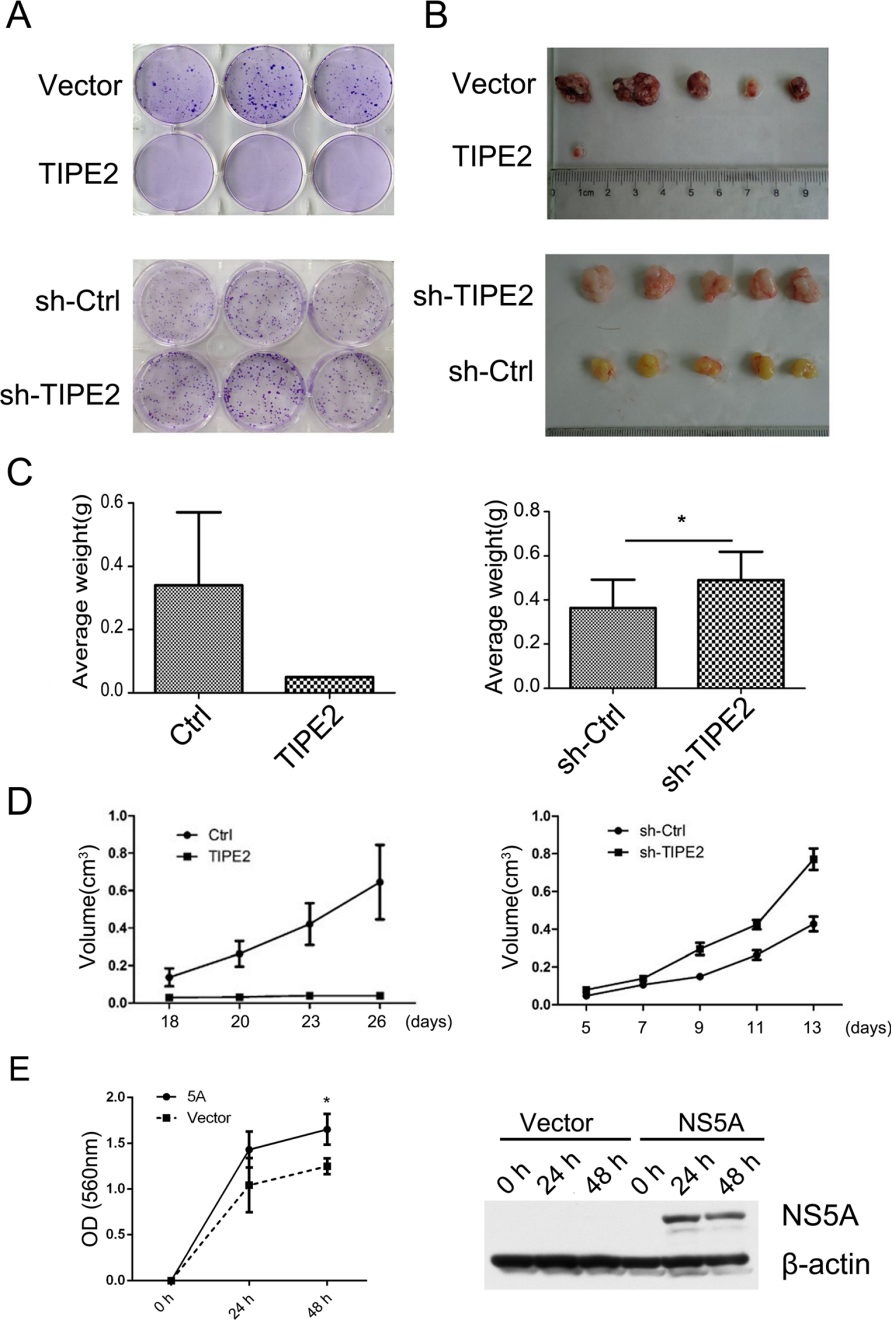
TIPE2 suppresses hepatocellular tumorigenesis in vitro and in vivo. **a** TIPE2-overexpressing, TIPE2 silencing, and control cells were utilized for the plate colony formation assay, respectively. **b** Isolated tumors from mice injected with control, TIPE2-overexpressing, or TIPE2 silencing cells. **c** The average weights of isolated tumors in **b** were quantified. **d** The growth curves of subcutaneous tumors from the mice. **e** Cell viability was measured up to 48 h by MTT assay in TIPE2 stable expressing Huh7 cells after transient transfection with NS5A and control plasmids. *p < 0.05

To examine the potential roles of TIPE2 in the inhibition of HCC tumor growth, we performed plate colony formation assay using Huh7-TIPE2 overexpression and Changliver-TIPE2 knockdown stable cell lines in vitro. As shown in Fig. 6a, almost no clone was formed by the Huh7-TIPE2-overexpressing cells. However, colony formation efficiency of Changliver-TIPE2 knockdown cells was significantly higher than that of the control cells. The results of Huh7-TIPE2 and Changliver-TIPE2 knockdown xenografts in nude mice suggest that the tumor volume and growth speed of Huh7-TIPE2 overexpression cells are much reduced compared to Huh7 itself (Fig. 6b–d). In contrast, the tumor growth and tumor volume derived from the Changliver-TIPE2 knockdown cells are much increased compared to the tumors of Changliver cells itself (Fig. 6b–d). Furthermore, we also observed the higher viability in TIPE2 stable Huh7 cells after NS5A overexpression (Fig. 6e). These results suggest that TIPE2 may negatively regulate HCC tumorigenesis.

### TIPE2 inhibits DDR in tumor

**Fig. 7:**
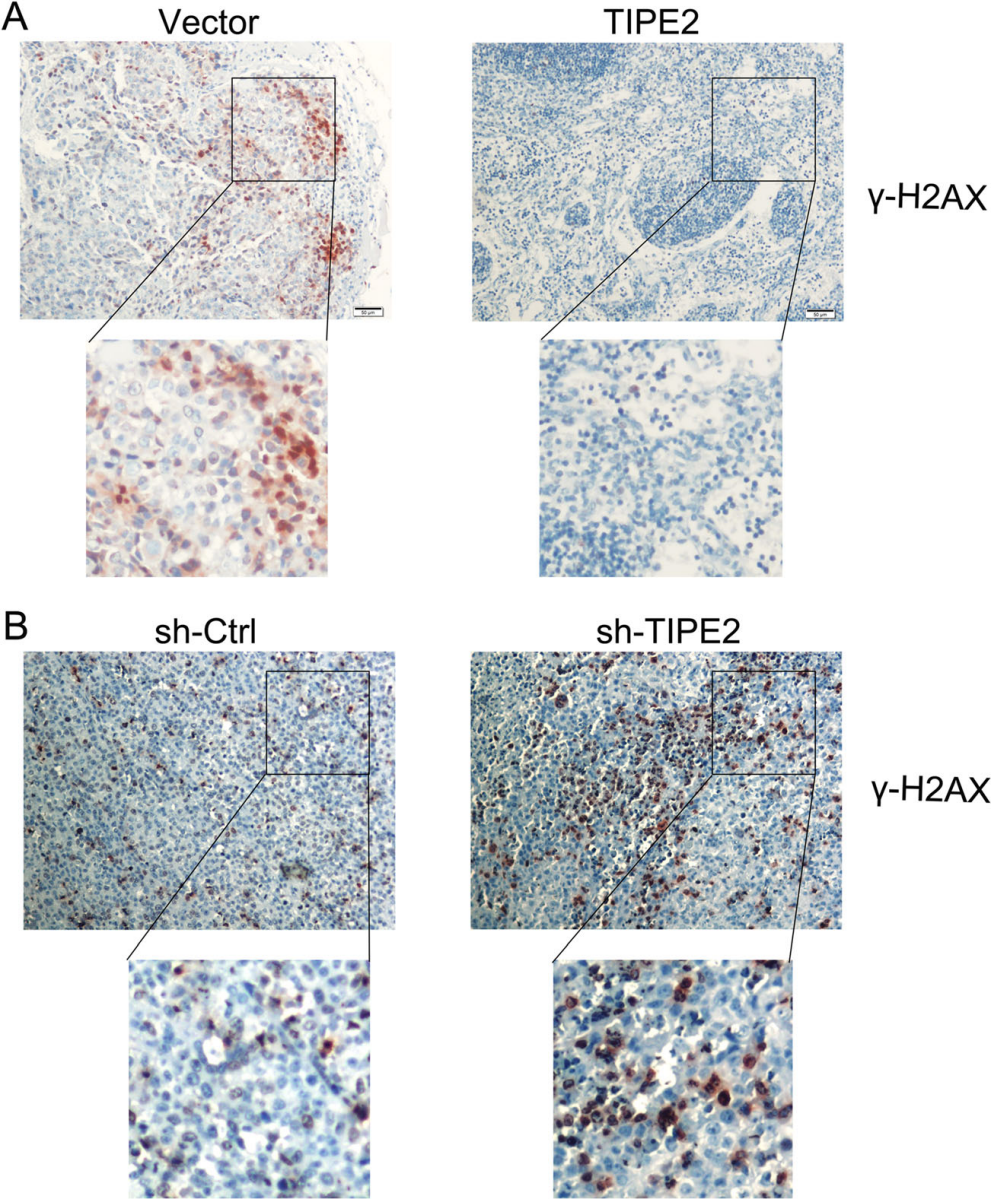
DNA damage in tumor tissues generated in the nude mice. Expression of γ-H2AX in the tumor tissues isolated from the mice injected with Huh7-TIPE2 and control cells (**a**) or Changliver sh-TIPE2 and control cells (**b**). Positive cells were stained in *red* in the nuclei. *Scale bars* = 50 μm

To determine the molecular mechanism underlying the TIPE2 effect, we studied DNA damage response (DDR) in the tumors derived from the nude mice by immunohistochemistry. As shown in Fig. 7a, the expression of γ-H2AX is much higher in the tumor tissue generated from Huh7 cells (Huh7-vector) than that in the Huh7-TIPE2 tumor tissues. In contrast, the immunohistochemistry staining of γ-H2AX is much stronger in Changliver-TIPE2 knockdown tumors than that in the tumors generated from parental Changliver cell (Fig. 7b). These results strongly suggest that TIPE2 can inhibit tumorigenesis by suppressing the DNA damage response.

## Discussion

TIPE2 is a newly identified regulator of immunity, while emerging evidence indicates that TIPE2 might be also a novel tumor suppressor. We therefore hypothesized that TIPE2 might be employed by cancer-causing pathogens, such as HBV and HCV, to initiate tumorigenesis or promote tumor growth. Intriguingly, recent studies have reported that TIPE2 expression was decreased in the peripheral blood mononuclear cells (PBMCs) of patients with chronic hepatitis B and in HBV-related HCC tumor tissue [21, 23]. Compared to HBV, HCV infection is more strongly associated with cirrhosis, hepatic decompensation, and HCC [1]. In the present study, for the first time, we observed downregulation of TIPE2 in the HCV-positive HCC tissue from a cohort of patients. These data suggest that TIPE2 is a common pathway of virus-induced HCC development.

While downregulation of TIPE2 was noticed in HCC tissue, the mechanisms underlying it remain unknown. In the current study, we found that one of the HCV-encoded non-structural proteins NS5A was adversely associated with the expression of TIPE2. This suggests that NS5A leads to degradation of TIPE2. There are mainly two pathways leading to protein downregulation: transcriptional regulation and protein modification. Interestingly, no significant difference was observed at mRNA level of TIPE2 between HBV-related HCC and its adjacent non-tumor tissue, while obviously higher level of ubiquitination of TIPE2 was detected and the proteasome inhibitor MG132 could restore TIPE2 protein accumulation [18]. Those data suggest that TIPE2 was degraded at protein level in HCC. We also found that TIPE2 could be restored by MG132 at the presence of NS5A. The mechanism by which NS5A induces the degradation of TIPE2 is not clear and needs further investigation.

Reactive oxygen species (ROS) and the products of inducible nitric oxide synthase (NOS) generated upon HCV infection could lead to DNA damage [9, 24]. HCV-encoded core, NS2, NS3/4A, and NS5B have been reported to trigger DNA damage. There have been also several studies that revealed association between NS5A and genomic instability via ROS. High level of ROS could be induced by NS5A in transgenic mice, cooperated with upregulation of NF-κB and STAT3, which promote steatosis and HCC [25]. NS5A also reduced tumor suppressor PTEN expression in ROS-dependent pathway [26]. In fact, NS5A is associated with chromosomal instability and mitotic cell cycle dysregulation [10]. Interestingly, NS5A was recently shown to downregulate the growth arrest and DNA damage-inducible gene 45-α (GADD45α) expression [27]. In this study, we showed that NS5A reduced TIPE2 expression and consequently induced DNA damage response. Activation of DNA damage response may result in impairment of damage repair, dysregulation of cell cycle, apoptosis, or tumor formation. Previous investigation has demonstrated that tumor is developed spontaneously in NS5A transgenic mice [28]. Our findings provide further evidence and molecular mechanism for the oncogenic effect of NS5A.

The connection between TIPE2 and DNA damage is surmisable according to the existing data. During infection, TIPE2 could inhibit phagocytosis and oxidative burst by binding to and blocking Rac GTPases [22]. Rac was reported to stimulate DDR. Furthermore, Rac1 was suggested responsible for the genotoxin-induced DNA damage in the liver [29]. On the other hand, Rac-mediated nuclear mechanisms are required for activation of DDR following topo II poison challenge in a p53- and heat shock protein-independent pathway [30]. So we predict that the upregulation of γ-H2AX, pChk1, and pChk2 in TIPE2 deficiency cells and HCC tumor tissue indicates that TIPE2 inhibits DDR during virus infection likely via the Rac pathway. Taken together, we propose that TIPE2 is centralized in a pathogenic network of infection, inflammation, DNA damage, and tumor formation.

In summary, our work suggests that HCV promotes HCC development via DNA damage, through downregulation of TIPE2 by its encoded NS5A protein. Our study provides a novel mechanism underlying HCV-related HCC development and identifies TIPE2 as a potential therapeutic target for HCV-related HCC.

## Abbreviations

HCC: Hepatocellular carcinoma
HCV: Hepatitis C virus
DDR: DNA damage response
TIPE2: Tumor necrosis factor-α-induced protein-8 like-2
ROS: Reactive oxidative stress
NOS: Nitric oxide synthase
